# The co-occurrence of mental disorders among Dutch adolescents admitted for acute alcohol intoxication

**DOI:** 10.1007/s00431-020-03823-0

**Published:** 2020-10-06

**Authors:** Loes de Veld, Joris J. van Hoof, Inge M. Wolberink, Nicolaas van der Lely

**Affiliations:** 1 Department of Paediatrics, Reinier de Graaf Gasthuis, Reinier de Graafweg 5, 2635 AD Delft, The Netherlands; 2 Faculty of Behavioural, Management and Social Sciences, University of Twente, PO Box 217, 7500 AE Enschede, The Netherlands

**Keywords:** Adolescents, Acute alcohol intoxication, Mental disorders, Neuropsychological assessment, ADHD

## Abstract

Adolescents with substance use disorders are often diagnosed with co-occurring mental disorders. However, it is unknown if adolescent hospital admission for acute alcohol intoxication is also associated with co-occurring mental disorders. Therefore, the primary aim of this study is to estimate the prevalence of co-occurring mental disorders among Dutch adolescents admitted for acute alcohol intoxication. Secondly, this study aims to explore the cross-sectional relationship between the co-occurrence of mental disorders and patient characteristics, such as sex, age and blood alcohol concentration at admittance. Data were retrospectively collected from 726 adolescents admitted for acute alcohol intoxication. Overall, 245 (34%) of the 726 adolescents treated for acute alcohol intoxication were diagnosed with a co-occurring mental health disorder, such as attention-deficit hyperactivity disorder (13%) or autism spectrum disorder (2.1%). Attention-deficit hyperactivity disorder in particular seems to be more prevalent in the study population than in the general Dutch adolescent population.

*Conclusion*: This study demonstrates that among adolescents admitted for acute alcohol intoxication, the prevalence of co-occurring mental disorders is a common and a relevant issue for treatment and prevention strategies.

**Table:** 

**What is Known:** *• Alcohol consumption among adolescents has been associated with negative psychosocial effect*. *• Among adolescents admitted for acute alcohol intoxication, risk factors for psychological dysfunction appear to be inadequately assessed, documented and followed up.*	
**What is New:** *• The current study reports on the prevalence of co-occurring mental disorders among a substantial sample of adolescents admitted for acute alcohol intoxication.* *• Understanding the prevalence of co-occurring mental disorders is clinically relevant for the outpatient follow-up of adolescents admitted for acute alcohol intoxication.*

**What is Known:**

*• Alcohol consumption among adolescents has been associated with negative psychosocial effect*.

*• Among adolescents admitted for acute alcohol intoxication, risk factors for psychological dysfunction appear to be inadequately assessed, documented and followed up.*

**What is New:**

*• The current study reports on the prevalence of co-occurring mental disorders among a substantial sample of adolescents admitted for acute alcohol intoxication.*

*• Understanding the prevalence of co-occurring mental disorders is clinically relevant for the outpatient follow-up of adolescents admitted for acute alcohol intoxication.*

## Introduction

Detecting the possible co-occurrence of mental disorders among adolescents admitted for acute alcohol intoxication is primarily necessary to treat each individual patient to the best extent possible. The detection of co-occurring mental disorders also aims at the prevention of repeated hospital admissions and the prevention of regular alcohol consumption in later life. Prevention of alcohol use in later life is important because a recent study noted that alcohol use of > 100 g per week at the age of 40 not only is a causal factor in many diseases but also increases all-cause mortality [1].

According to the global status report on alcohol and health 2018 published by the World Health Organization, more than half of the European population aged 15–19 years has used or is currently using alcohol. With regard to patterns of alcohol consumption, heavy episodic drinking among young people aged 15–19 years is particularly prevalent in Europe and high-income countries such as Australia, Canada and the United States [2]. From an international perspective, studying adolescent alcohol use in the Netherlands is an important case study as during the last decade a discrepancy has been observed between the declining trend of regular alcohol use in the Dutch adolescent population [3] and the rising trend of hospital admissions for alcohol intoxication on the other [4].

At admission for acute alcohol intoxication, the presence of other mental disorders should be considered. Prior research showed an association between alcohol exposure and mental disorders [5, 6]. For adolescents, initiation of alcohol usage has been correlated with mental disorders; symptoms of depression were more prevalent in adolescents who had tried alcohol compared with adolescents who never tried alcohol [7]. Although these studies indisputably associate alcohol misuse among adolescents with negative psychosocial effects, studies about the psychosocial consequences of single episodes of alcohol intoxication are scarce.

This might be explained by research that suggests that risk factors for psychological dysfunction among adolescents admitted for acute alcohol intoxication appear to be inadequately assessed and documented [8]. A recent study among 40 adolescents with acute alcohol intoxication did not find differences in psychosocial well-being and health-related quality of life to match controls. However, due to the sample size, it remains unclear whether significant relations are absent or untraceable. [9]

Understanding the prevalence and patterns of co-occurring mental health disorders among adolescents admitted for acute alcohol intoxication could assist paediatricians in organizing appropriate outpatient follow-up care with the aim of reducing repetitive admissions. In the current study, medical records of 726 Dutch adolescents were analysed with the prior aim of establishing an estimate of the prevalence of co-occurring mental disorders in the population of adolescents admitted for acute alcohol intoxication in contrast to the general Dutch adolescent population.

## Method

### Study design and study population

This retrospective observational study was conducted in the Reinier de Graaf hospital, Delft, the Netherlands, where in 2007 a prevention-intervention program at the ‘Outpatient Department for Adolescents and Alcohol’ was implemented for adolescents with acute alcohol intoxication [10]. Participants were selected using the diagnosis and treatment combination code ‘intoxication’, which is used by all Dutch hospitals to register and declare expenses related to intoxication. All patients aged 10–18 with alcohol intoxication were manually included. Patients not invited for the program were excluded from the analysis.

### Data collection

All data were extracted from two data sources: the registration form for report to the Dutch Paediatric Surveillance System (NSCK) form for measures related to the acute alcohol intoxication event and electronic medical records for measures related to the follow-up of patients with acute alcohol intoxication. Since 2007, the NSCK has been collecting data of adolescence admitted for acute alcohol intoxication, such as demographic characteristics, intoxication characteristics and substance use patterns. The requirement for inclusion for this study was the written consent of adolescents (and his/her parents if the adolescent was younger than 16 years of age) for reporting to the Dutch Paediatric Surveillance System. As the NSCK registration form does not contain details on outpatient follow-up, additional data were extracted from electronic patient records.

To determine the prevalence of mental disorders among adolescents invited to participate in the prevention-intervention program, first, the process at the ‘Outpatient Department of Adolescents and Alcohol’ was evaluated. The program consisted of four consecutive stages: invitation to the program (1), consultation with a paediatrician aimed at reflecting on the alcohol intoxication incident (2), a screening consultation with the child psychologist aimed at identifying risk factors for binge drinking and symptoms of underlying neuropsychological disorders (3) and neuropsychological assessment if the screening consult revealed an indication for further research (4). Flowchart 1 represents the different stages of the program. Adolescents could quit the program at any stage.

**Flowchart 1:**
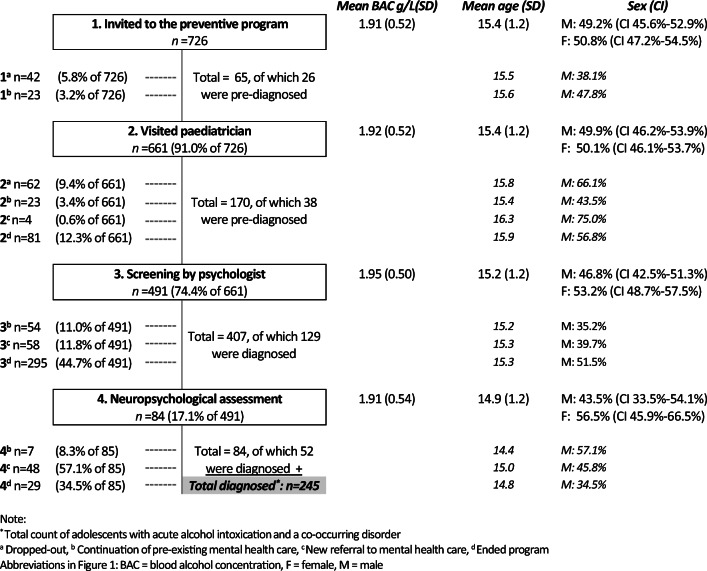
*Frequency of neuropsychological assessment and co-occurring mental health disorders.* Note: *Total count of adolescents with acute alcohol intoxication and a co-occurring disorder. (a) Dropped out. (b) Continuation of pre-existing mental healthcare. (c) New referral to mental healthcare. (d) Ended program. Abbreviations in Fig. 1: BAC, blood alcohol concentration; F, female; M, male

Secondly, a standardized abstraction form was used to systematically screen different sections within the electronic health records, namely, past medical history, medication, medical records on date of emergency department visit for acute alcohol intoxication, medical records on visits to the ‘Outpatient Department for Adolescents and Alcohol’ and the outpatient letter by the child psychologist. Each subcategory of co-occurring mental disorders was registered as a binary categorical variable: attention-deficit (hyperactivity) disorder (ADD/ADHD); autism spectrum disorder (ASD); anxiety disorder (ANX); trauma- and stressor-related disorder (TRAUMA); disruptive, impulse-control and conduct disorders (DIC); substance use disorders (SUD); depressive disorders (DD) and other unspecified mental health disorder. Other unspecified mental health disorders were recorded as a string variable and consisted of a heterogeneous group of disorders. In order to prevent traceability to a person, this subgroup will not be specified any further. Data were extracted by two of the researchers independently and any discrepancies in coding were reviewed jointly and discussed to improve reliability. Discrepancies were reconciled by third party resolution.

Co-occurring mental disorders were either diagnosed prior to the hospital admission for acute alcohol intoxication or diagnosed during the follow-up program. Pre-diagnosed co-occurring disorders were derived from medical history. As the diagnostics were performed in specialist mental healthcare institutions, the process of diagnosis is unknown. Pre-diagnosed disorders were verified by either the general practitioner or the external practitioner of mental healthcare institution.

The coding of mental health disorders during the program was based on the outpatient letter from the child psychologist to the paediatrician containing the results of the psychological screening and if applicable and the summary statement about the results of neuropsychological assessment. The screening consultation with the child psychologist consisted of a semi-structured interview and the Child Behaviour Checklist (CBCL) [11], a validated instrument for identification of problem behaviour in adolescents. When indicated, neuropsychological assessment was performed using standardized tests for multiple cognitive functions: intelligence (Wechsler Intelligence Scale for Children), short- and long-term verbal memory (15 Words Test and Rey’s Visual Design Learning Task), concentration (Amsterdam Neuropsychological Task), cognitive flexibility and inhibition (Stroop Colour Test and Wisconsin Card Sorting Test), personality traits (Dutch Personality Questionnaire), coping styles (Utrecht Coping List) and ego development (Washington University Sentence Completion Test).

### Ethical approval and consent

The study protocol was approved by the Medical Ethical Research Committee Zuid Holland West (ref: 19-080). Partial waiver of informed consent for data extraction from electronic patient records was approved by the medical ethical research committee as the research could not practicably be carried out without the waiver as subjects, especially those age > 16 at the moment of admission for acute alcohol intoxication, could be burdened by using the contact information in the medical records, as parents might not have been informed due to the medical confidentiality regulations. However, similar inclusion criteria (adolescents < 18 years, BAC > 0.0) have been adhered by the NSCK for research to acute alcohol intoxication and therefore consent for this study was deduced from the NSCK registration form. Adolescents (and in the case of adolescents < 16 years of age parents) that did not provide written consent for NSCK registration were excluded from the study in step 1A; only age and sex were registered and their health records were not screened for coding (Flowchart 1).

For the interpretation of the study results, it is important to realize that in the hospital where this study was performed, the department of clinical paediatric psychology is independent from the paediatric department. Due to local policies and privacy measures, the patients’ records and specific test results are registered in a restricted area of the electronic health records not accessible for paediatricians and doctors. Patients of the child psychology department provide informed consent for the report of the results of the screening consult and if applicable neuropsychological assessment to the paediatrician. In case of a missing outpatient letter, the other sections of the medical record were decisive. If the other sections were negative for a co-occurring mental disorders, patients were included in the category ‘absence of mental disorders’, even though this methodology might cause underrepresentation of the prevalence of co-occurring mental disorders.

### Statistical analyses

The IBM SPSS Statistics for Windows, version 25.0 (released 2017 by IBM Corp, Armonk, NY), was used to analyse the data. Proportions were expressed as percentages, with 95% confidence intervals (CIs). All continuous data were expressed as the average with standard deviation (SD).

In the determination of the prevalence of mental disorders, adolescents that dropped out before the first visit to the paediatrician could be considered missing data, as follow-up might have resulted in a diagnosis. In order to prevent overestimation of the prevalence of mental health disorders, these study participants were not excluded from analysis, but the medical records on the date of emergency department presentation were used for coding. The prevalence of mental disorders in the study population was compared with the prevalence of mental disorders in a sample that was representative of the Dutch adolescent population with a mean age of 19.1 years old: the TRacking Adolescents’ Individual Lives Survey (TRAILS) sample [12] using binomial z-tests.

Within the study population, adolescents with a co-occurring mental disorder were compared with the control group without co-occurring mental disorder on several outcome parameters: sex, educational level, family structure, ethnicity, age at first alcohol use, age at admission and BAC. The association between categorical variables and the presence of co-occurring mental disorders were determined using Pearson’s chi-square test. For each continuous variable, normality was assessed using the Kolmogorov-Smirnov test and depending on the results either an independent sample *t* test or a Mann-Whitney U test was performed. The association between co-occurring mental disorders and continuous variables was corrected for covariates by a multilinear regression model. The significance level of all these tests was set to *p* = .05. Descriptive statistics were used to display qualitative differences in patient characteristics between the earlier mentioned subcategories of mental health disorders. Due to expected small group sizes and the risk of multiple testing, no separate statistical analyses have been performed on these subcategories.

## Results

### Study population and dropout analyses

During the period 2007–2017, 747 individual adolescents received the diagnosis and treatment combination code ‘intoxication’ for an alcohol intoxication. Of these 747 adolescents, 726 were invited for the prevention-intervention program at the ‘Outpatient Department for Adolescents and Alcohol’ and included in the current study population. The exclusion of 21 adolescents was either due to deviation from protocol (*n* = 12) or referral for follow-up to province/country of origin (*n* = 9).

The mean age of the study population was 15.4 years and the study population consisted of slightly more female adolescents (50.1%) than male adolescents (49.9%). Four different reasons for discontinuation were noted: dropping out (A), continuation of care at other healthcare institutions (B), new referral to mental healthcare (C) or completion of the program (D). The group that dropped out directly after invitation (1A) consisted of 2 categories: no written consent for NSCK registration (*n* = 15) and not showing up at/cancellation of appointment with paediatrician (*n* = 27).

As displayed in Flowchart 1, the total number of adolescents who were already mental healthcare clients prior to the preventive program (total of category B) was 108 (15%, CI 12–18%). During the program, 109 adolescents (15%, CI 12–18%) were referred to mental healthcare or addiction care (category C). Overall, among the adolescents invited for the program, 217 (30%, CI 27–33%) received a form of mental healthcare prior to the acute alcohol intoxication or were referred to a mental healthcare institution after the intoxication.

### Prevalence of co-occurring mental disorders

As displayed in the grey box in Flowchart 1, overall, 245 out of 726 (34%, CI 30%–37%) adolescents were diagnosed with a co-occurring mental health disorder. The prevalence of mental disorders in the study does not differ from the prevalence in the TRAILS sample.

ADHD was the most frequently co-occurring mental disorder in the study population with a prevalence of 13% (61 with ADHD and 33 diagnosed with multiple co-occurring mental disorders including ADHD). A binomial z-test indicated that the prevalence of ADHD was higher among the study population than in the reference population. The prevalence of ASD in the study population was 2.1% out of 727 (6 with ASD and 9 with multiple comorbidities including ASD). The TRAILS study did not assess ASD.

Both the 12-month prevalence of DD and the 12-month prevalence of ANX were lower in the study population than in the reference population. There were no differences between the study population and the TRAILS sample for the prevalence of DIC and for SUD. The prevalence of TRAUMA in the study population was 2.1%, and the TRAILS study did not research the prevalence of TRAUMA.

Out of 245 adolescents with co-occurring mental disorders, 46 adolescents were diagnosed with multiple co-occurring mental disorders. Of these 46, most were diagnosed with ADHD and one or more other mental disorders (*n* = 33).

### Relationship between the co-occurrence of mental disorders and acute alcohol intoxication characteristics

Table 1 displays the patient characteristics of adolescents with versus adolescent without co-occurring mental health disorder. The proportion of females was significantly higher in adolescents with co-occurring mental disorders (50.8%) than in adolescents without co-occurring mental disorders (48.2%). Pearson’s chi-square test indicated that the presence of co-occurring mental disorders is sex-related (χ2(1, *n* = 726) = 3.84, *p* < .05). The educational level was significantly higher among adolescents without a co-occurring mental disorder than in adolescent with a mental disorder (χ2(3, *n* = 650) = 18.72, *p* < .05). Furthermore, adolescents with a co-occurring mental disorder were more frequently raised in a non-traditional family structure (47.3%) than adolescents without a co-occurring mental disorder (25.0%), χ2(1, *n* = 566) = 27.52, *p* < .05. Although the proportion of cases in which a drug screening was performed did not differ significantly between those with a co-occurring mental disorder and the reference group, a positive screening was found more frequently in adolescents with co-occurring mental disorder, compared with those without a co-occurring mental disorder (Tables 2 and 3).

**Table 1:** Prevalence of mental disorders among adolescent with alcohol intoxication

Mental disorder	Prevalence in study population (*n* = 726)		Prevalence in TRAIL sample (*n* = 1584)	Binomial z-test
Any mental disorder	*n = 245*	33.7% (CI 30.7–37.3%)	31.0%	2P (*Y* = 245|*n* = 726, *p* = 0.310) = 0.06
*ADHD*	*n* = 94	12.8% (CI 10.6–15.7%)	3.2%	2P (*Y* = 94|*n* = 726, *p* = .032) = **< 0.001**
*ASD*	n = 15	2.1% (CI 1.3–2.1%)	–	–
*DD*	*n = 42*	5.8% (CI 4.3–7.8%)	8.8%	2P (*Y* = 15|*n* = 726, *p* = .088) = 0**.002**
ANX	*n = 5*	0.7% (CI 0.3–1.6%)	18.4%	2P (*Y* = 5|*n* = 726, *p* = .184) = **<0.001**
TRAUMA	*n = 15*	2.1% (CI 1.3–2.1%)	–	–
DIC	*n = 27*	3.7% (CI 2.5–5.4%)	4.2%	2P (*Y* = 27|*n* = 726, *p* = .042) = 0.30
SUD	*n = 29*	4.0% (CI 2.7–5.8%)	4.9%	2P (*Y* = 29|*n* = 726, *p* = .049) = 0.15

*ADHD* attention-deficit hyperactivity disorder, *ANX* anxiety disorder, *ASD* autism spectrum disorder, *DD* depressive disorder, *DIC* disruptive, impulse-control and conduct disorder, *SUD* substance use disorder, *TRAUMA* trauma- and stressor-related disorder. Hyphen indicates that the prevalence has not been assessed in the TRAIL sample and that therefore no binominal z-test has been performed

**Table 2:** Acute alcohol intoxication characteristics

Characteristics	Acute alcohol intoxication without co-occurring mental disorder *n* = 481	Acute alcohol intoxication with co-occurring mental disorder *n* = 245	Statistics
Sex	*(n = 481)*	*(n = 245)*	*p* = 0.05^a^
Male	51.8% (CI 47.2–56.3%)	44.1% (CI 37.8–50.5%)	
Female	48.2% (CI 43.7–52.8%)	55.9% (CI 49.5–62.5%)	
Educational level	(*n* = 436)	*(n* = 214)	*p* < 0.001^a^
Low	36.0% (CI 31.5–40.7%)	48.6% (CI 41.8–55.5%)	
Middle	31.2% (CI 26.9–35.8%)	20.6% (CI 15.5–27.7%)	
High	22.7% (CI 18.9–27.0%)	15.4% (CI 11.0–21.1%)	
Different type	10.1% (CI 7.5–13.4%)	15.4% (CI 11.0–21.1%)	
Ethnicity	*(n = 395)*	(*n* = 198)	*p* = 0.56^a^
Dutch	84.6% (CI 80.5–87.9%)	86.4% (CI 80.6–90.6%)	
Different ethnicity	15.4% (CI 12.1–19.5%)	13.6% (CI 8.4–19.4%)	
Family structure	*(n = 376)*	(*n* = 190)	*p* < 0.001^a^
Traditional family structure	75.0% (CI 70.3–79.2%)	53.2% (CI 45.8–60.4%)	
Non-traditional family structure	25.0% (CI 20.8–29.7%)	47.2% (CI 39.6–54.2%)	
Age at first alcohol use	*(n = 323)*	(*n* = 160)	*p <* 0*.001*^*b*^
	14.41 years (SD 1.38)	13.85 years (SD 1.66)	
Age at admission (intoxication)	(*n* = 481)	*(n = 245)*	*p* = 0.21^b^
	15.41 years (SD 1.19)	15.29 years (SD 1.24)	
Blood alcohol concentration	(*n* = 462)	*(n =* 234*)*	*p* = 0.03^b^
	1.94 g/L (SD 0.50)	1.85 g/L (SD 0.56)	
Illicit drug use (% performed)	(*n* = 481)	(n = 245)	*p =* 0*.40*^b^
Drug screening performed	57.6% (CI 52.8–61.8%)	60.8% (CI 54.4–66.9%)	
No drug screening performed	42.4% (CI 38.0–47.0%)	39.2% (CI 33.1–45.6%)	
Result drug screening	(*n* = 277)	(*n* = 149)	*p <* 0*.001*^*b*^
Positive drug screening	92.4% (CI 88.5–95.1%)	75.2% (CI 67.3–81.7%)	
Negative drug screening	7.6% (CI 4.9–11.5%)	24.8% (CI 28.3–32.7%)	
Subtypes positive drug screening	*n* = 21	*n = 37*	
Cannabis	*n* = 16	*n* = 18	
(Meth)amphetamines	*n* = 2	*n* = 5	
Cocaine	*n* = 0	*n* = 3	
Different	*n* = 2	*n* = 5	
Multiple	*n* = 1	*n* = 6	

a = chi-square test. b = Mann-Whitney U test

**Table 3:** Relationship between the co-occurrence of mental disorders and acute alcohol intoxication characteristics

Mental disorder	Count and percentage	Sex distribution	Age in year	BAC g/L
Acute alcohol intoxication without co-occurring mental disorder	*n* = 481 (66.4%, CI 62.7–69.6%)	♂51.8–♀48.2%^*^	15.41 (1.18)	1.94 (0.50)^*^
Acute alcohol intoxication with co-occurring mental disorder	*n* = 245 (33.8%, CI 30.4–37.2%)	♂44.1–♀55.9%^*^	15.29 (1.24)	1.85 (0.56)^*^
*ADHD*		♂54.1–♀45.9%	15.43 (1.30)	1.98 (0.58)
*ASD*		♂66.7–♀33.3%	14.83 (1.47)	2.02 (0.82)
*DD*		♂21.4–♀78.6%	14.79 (1.23)	1.80 (0.50)
*ANX*		♂0.0–♀100.0%	15.67 (1.53)	1.63 (0.21)
*TRAUMA*		♂22.2–♀77.8%	15.11 (1.45)	1.65 (0.46)
*DIC*		♂58.3–♀ 41.7%	14.58 (1.08)	1.62 (0.82)
*SUD*		♂57.1–♀ 42.9%	16.00 (0.96)	2.10 (0.76)
*OTHER*		♂30.8–♀69.2%	15.25 (1.16)	1.78 (0.52)
*Multiple mental disorders*		♂59.6–♀40.4%	15.53 (1.18)	1.81 (0.53)

^a^Pearson chi-square, *p* < 0.05. ^b^Mann-Whitney U test, not significant. ^c^Mann-Whitney U test, *p* < 0.05

*ADHD* attention-deficit hyperactivity disorder, *ANX* anxiety disorder, *ASD* autism spectrum disorder, *DD* depressive disorder, *DIC* disruptive, impulse-control and conduct disorder, *OTHER* other unspecified mental disorder, *SUD* substance use disorder, *TRAUMA* trauma- and stressor-related disorder

The descriptive statistics for the subcategories are displayed in table. Adolescents with co-occurring mental disorders were younger at the age of first alcohol use than adolescents without a co-occurring disorder. After correction for the covariates sex, educational level, ethnicity and family structure by linear regression analysis, the association between the presence of co-occurring mental disorders and age at first alcohol use remained significant (*p* < .01). The association between co-occurring mental disorders and BAC became insignificant after correction for co-occurring mental disorders (*p* = .10).

## Discussion

The current study reports the prevalence of co-occurring mental disorders among a substantial sample of adolescents admitted for acute alcohol intoxication. The purpose of the comparison between the highly selective study population (with a mean age of 15.4) and the TRAILS sample (mean age 19.1 years old) was to set a frame of reference for clinicians. Although some of the prevalence differences might be partly attributable to this age difference, setting a frame of reference was considered more important than the risk of bias. Preferably, the reference population or a control group would have been similar in mean age and representative of the general Dutch adolescent population.

Selection of a reference group representative of the general Dutch adolescent population with such a specific mean age is challenging and as far as the authors know, Dutch studies studying the prevalence of mental health disorders in adolescents are scarce. Although the age difference between the 2 populations provokes challenges and bias, associations between age and the prevalence of mental health disorders are relatively well explored. For example, a group of matched controls within the hospital setting (e.g. age-matched adolescents at the outpatient department or emergency department) would result in other confounding factors which might be harder to interpret than age. Furthermore, the TRAILS sample displays standardized cumulative prevalence graphs by age, which help to explain the differences between the study population and reference population attributable to age. Although the comparison might not be relevant from an epidemiological perspective, the comparison is relevant from a clinical paediatric perspective as it might help clinicians involved in the acute care for adolescents with acute alcohol intoxication to value the prevalence of co-occurring mental disorders in the study population and assist them in organizing appropriate follow-up. The relevance of the age differences will be assessed later in this discussion for various mental health disorders.

Overall, 33.8% of the adolescents admitted for acute alcohol intoxication had at least one co-occurring mental disorder, and 29.9% of the adolescents invited to participate in the program had an indication for mental healthcare or care by youth social services. This is in line with several prior studies that suggest that the prevalence of mental health disorders [13, 14] and social problems is elevated among young adults admitted for alcohol intoxication [15, 16].

Acute alcohol intoxication and the presence of concurrent mental disorders were sex-specific and occurred more frequently in girls than in boys. This is in line with prior research. Women with SUD have a significantly higher prevalence of comorbid psychiatric disorders than men [17]. Another explanation might be that girls are considered to be more vulnerable to the neurotoxic effects of binge drinking during adolescence [18]. The presence of co-occurring mental disorders was significantly associated with a younger age at first alcohol use, which is worrisome since age at first alcohol use has frequently been associated with increased alcohol consumption later in life [19, 20]. As the prevalence of co-occurring mental disorders among adolescents admitted for acute alcohol intoxication is substantial, prevention of acute alcohol intoxication might be improved by the identification of substance use among adolescents, especially those with a mental health disorder. A recent article provided interview tools that can assist primary care providers efficiently to address problematic substance use by adolescents [21].

The prevalence of ADHD in the study population was significantly higher than the prevalence of ADHD in the TRAILS sample. This result can be partially explained by a higher mean age of adolescents in the TRAILS sample compared with the study population, as prior epidemiological studies with a large sample size found a negative association between age and prevalence of ADHD [22, 23]. The cumulative prevalence graphs in the TRAILS study indicate that ADHD occurred earliest in childhood, with virtually no new onset after the age of 6 years [12]. The overrepresentation of ADHD in the study population is in line with prior research that indicated that adolescents with ADHD were significantly more likely to develop SUD than adolescents without ADHD [24]. Research has also shown that among adults with a SUD, 23% also meet the criteria for ADHD, and even impulsivity facets without the diagnosis of ADHD were related to a higher prevalence of alcohol disorders [25]. The results of our study support a recent modified Delphi study in which a multidisciplinary group of 55 experts from 17 countries agree on the statement that routine screening for ADHD is recommended in substance abuse treatment [26].

The prevalence of ASD in the study population was 2.1%, while a recent meta-analysis reported a global prevalence of ASD of 0.7 per 100 [27].This might suggest an overrepresentation of adolescents with ASD in the study population, which is in contrast to a prior study that shows that elevated autistic trait scores were not significantly associated with adolescent alcohol use and misuse [28].Therefore, an overrepresentation of ASD in the study may indicate that in the case of engagement, adolescents with ASD seem to be more vulnerable to binge drinking resulting in acute alcohol intoxication.

Prior research shows that depressive symptoms are associated with harmful use of alcohol [6, 29]. However, the prevalence of DD was significantly lower in the study population than in the TRAILS population. This might be the result of a lower mean age of the study population in comparison with the TRAILS sample. The TRAILS study shows that mood disorders were not prevalent until early adolescence, after which their incidence rose steadily. Dutch national statistics indicate that the self-reported half-year prevalence for depression is higher among older adolescents. In 2017, the self-reported 12-month prevalence of DD of 12- to 16-year-old adolescents was 3.1%, while the self-reported 12-month prevalence of adolescents older than 16 years of age is 10.2% [30].

The prevalence of co-occurring ANX disorders in the study population was low in comparison with the TRAILS sample in which the most common ANX disorder was specific phobia. Specific phobias might be missed in the screening of the psychologist in the follow-up for acute alcohol intoxication patients. In research among adults diagnosed with both alcohol dependence and a social phobia, drinking alcohol eventually became unable to alleviate social phobia symptoms or worsened symptoms [31]. A Finnish study among adolescents revealed that comorbid general anxiety increased the persistence of frequent alcohol use while comorbid symptoms of social phobia decreased its persistence [32].

The prevalence of TRAUMA in the study population was 2.1%. Prior research among pre-adolescent children 10–13 years old indicated that PTSD symptoms may be associated with early onset of alcohol use [33]. Several studies indicate that university students with trauma and, in particular, post-traumatic stress disorders are at elevated risk for a problematic drinking pattern [34–36].

The prevalence of DIC among adolescents invited for the prevention-intervention program did not differ significantly from the 12-month prevalence in the TRAILS sample. A recent prospective follow-up study indicated that conduct disorder was associated with elevated adjusted hazards for initiation of all substances, with comparatively greater hazard ratios of initiating illicit drug use than alcohol use, at the age of 15 [37].

The prevalence of co-occurring SUD in the study population was 4.0% and did not differ significantly from the prevalence of SUD in the TRAILS population. Therefore, it appears that a co-occurring SUD is not directly associated with an increased risk of hospital admission for acute alcohol intoxication. The standardized cumulative prevalence graphs in the TRAILS study show that drug and alcohol dependence had the latest age of onset, with incidences beginning at the age of 14 years and steadily increasing after that [12]. Therefore, a non-significant comparison between the study population with a mean age of 15 years and the TRAILS sample with a mean age of 19 years might suggest that adolescents admitted for acute alcohol intoxication are at risk of an early SUD, which is in line with prior research which has shown that adolescents younger than 15 years of age who consume alcohol have a 4–6 times higher risk of developing alcohol dependence than adolescents who do not drink alcohol [38, 39].

The subcategory of other unspecified co-occurring mental disorders exists in a heterogeneous group from various Diagnostic and Statistical Manual of Mental Disorders (DSM) categories, for example, schizophrenia spectrum and other psychotic disorders, dissociative disorders, somatic symptom and related disorders, feeding and eating disorders or another unspecified mental health disorder. As each separate group consisted of only a few individuals per diagnosis (< 5), specification of the category OTHER would lead to traceable and identifiable patient information. The relatively low frequency (< 5) of schizophrenia spectrum and other psychotic disorders is an interesting result according to the DSM-5; between 7 and 25% of first-episode psychosis subjects have a substance-induced psychosis [40]. In a recent study, even a novel and separate clinical entity named as substance-related exogenous psychosis has been outlined [41]. However, the low prevalence of co-occurring psychotic disorders in the study population can be explained by both the study design and the organization of mental healthcare in the Netherlands. This specific study was focused on alcohol intoxication and although approximately 10% of the adolescents combined alcohol and illicit drug use, the numbers of illicit drug use were limited. Furthermore, if patients with a first episode of substance-induced psychosis have stable vital functions, patients will be assessed by the local crisis intervention team of specialized mental healthcare institutions. This might explain why adolescents with a first episode of substance-induced psychosis do not present in the hospital setting and therefore are underrepresented in the study population.

## Conclusion

This study indicated that the presence of mental disorders is common among adolescents admitted for acute alcohol intoxication. The descriptive statistics suggested that some of the subcategories seemed to be sex-specific or age-related. However, further research and an even larger study population are necessary in exploring these potential links further. Attention-deficit hyperactivity disorder in particular seems to be more prevalent in the study population than in the general Dutch adolescent population. This overrepresentation requires further research in the medical and psychological domains.

Co-occurrence of mental disorders is present among 34% of adolescents admitted for acute alcohol intoxication. Therefore, during admittance to the hospital, the social circumstances and signs of mental disorders should be considered. Follow-up of adolescents admitted for acute alcohol intoxication is necessary to prevent repeated admissions, to signalize mental disorders and to determine whether the patient requires referral to specialized mental healthcare. Neuropsychological assessment during follow-up of adolescents admitted for acute alcohol intoxication is indicated if screening provides signals for mental disorders. In our opinion, this assessment should take place in well-equipped centres with dedicated and professional specialized staff.

## Abbreviations

ANX: Anxiety disorder
ASD: Autism spectrum disorder
ADD/ADHD: Attention-deficit (hyperactivity) disorder
BAC: Blood alcohol concentration
DD: Depressive disorder
DIC: Disruptive, impulse-control and conduct disorders
DBC-codes: Diagnosis and treatment combination codes
SUD: Substance use disorder
TRAUMA: Trauma- and stressor-related disorder

## References

[1] Wood AM, Kaptoge S, Butterworth AS, Willeit P, Warnakula S, Bolton T, Paige E, Paul DS, Sweeting M, Burgess S, Bell S, Astle W, Stevens D, Koulman A, Selmer RM, Verschuren WMM, Sato S, Njølstad I, Woodward M, Salomaa V, Nordestgaard BG, Yeap BB, Fletcher A, Melander O, Kuller LH, Balkau B, Marmot M, Koenig W, Casiglia E, Cooper C, Arndt V, Franco OH, Wennberg P, Gallacher J, de la Cámara AG, Völzke H, Dahm CC, Dale CE, Bergmann MM, Crespo CJ, van der Schouw YT, Kaaks R, Simons LA, Lagiou P, Schoufour JD, Boer JMA, Key TJ, Rodriguez B, Moreno-Iribas C, Davidson KW, Taylor JO, Sacerdote C, Wallace RB, Quiros JR, Tumino R, Blazer DG, Linneberg A, Daimon M, Panico S, Howard B, Skeie G, Strandberg T, Weiderpass E, Nietert PJ, Psaty BM, Kromhout D, Salamanca-Fernandez E, Kiechl S, Krumholz HM, Grioni S, Palli D, Huerta JM, Price J, Sundström J, Arriola L, Arima H, Travis RC, Panagiotakos DB, Karakatsani A, Trichopoulou A, Kühn T, Grobbee DE, Barrett-Connor E, van Schoor N, Boeing H, Overvad K, Kauhanen J, Wareham N, Langenberg C, Forouhi N, Wennberg M, Després JP, Cushman M, Cooper JA, Rodriguez CJ, Sakurai M, Shaw JE, Knuiman M, Voortman T, Meisinger C, Tjønneland A, Brenner H, Palmieri L, Dallongeville J, Brunner EJ, Assmann G, Trevisan M, Gillum RF, Ford I, Sattar N, Lazo M, Thompson SG, Ferrari P, Leon DA, Smith GD, Peto R, Jackson R, Banks E, di Angelantonio E, Danesh J, Wood AM, Kaptoge S, Butterworth A, Willeit P, Warnakula S, Bolton T, Paige E, Paul DS, Sweeting M, Burgess S, Bell S, Astle W, Stevens D, Koulman A, Selmer RM, Verschuren M, Sato S, Njølstad I, Woodward M, Veikko S, Nordestgaard BG, Yeap BB, Flecther A, Melander O, Kuller LH, Balkau B, Marmot M, Koenig W, Casiglia E, Cooper C, Arndt V, Franco OH, Wennberg P, Gallacher J, Gómez de la Cámara A, Völzke H, Dahm CC, Dale CE, Bergmann M, Crespo C, van der Schouw YT, Kaaks R, Simons LA, Lagiou P, Schoufour JD, Boer JMA, Key TJ, Rodriguez B, Moreno-Iribas C, Davidson KW, Taylor JO, Sacerdote C, Wallace RB, Quiros JR, Rimm EB, Tumino R, Blazer III DG, Linneberg A, Daimon M, Panico S, Howard B, Skeie G, Salomaa V, Strandberg T, Weiderpass E, Nietert PJ, Psaty BM, Kromhout D, Salamanca-Fernandez E, Kiechl S, Krumholz HM, Grioni S, Palli D, Huerta JM, Price J, Sundström J, Arriola L, Arima H, Travis RC, Panagiotakos DB, Karakatsani A, Trichopoulou A, Kühn T, Grobbee DE, Barrett-Connor E, van Schoor N, Boeing H, Overvad K, Kauhanen J, Wareham N, Langenberg C, Forouhi N, Wennberg M, Després JP, Cushman M, Cooper JA, Rodriguez CJ, Sakurai M, Shaw JE, Knuiman M, Voortman T, Meisinger C, Tjønneland A, Brenner H, Palmieri L, Dallongeville JP, Brunner EJ, Assmann G, Trevisan M, Gillumn RF, Ford IF, Sattar N, Lazo M, Thompson S, Ferrari P, Leon DA, Davey Smith G, Peto R, Jackson R, Banks E, di Angelantonio E, Danesh J (2018). Risk thresholds for alcohol consumption: combined analysis of individual-participant data for 599 912 current drinkers in 83 prospective studies. Lancet.

[2] WHO, World Health Organization (2018) Global status report on alcohol and health 2018. World Health Organization. Available https://www.who.int/substance_abuse/publications/global_alcohol_report/gsr_2018/en/. Accessed May 14, 2019

[3] Van Laar MW, Van Ooyen-Houben MMJ, Meijers RF, Croes EA, Ketelaars APM, Van der Mol PM (2016) Nationale drug monitor – Jaarbericht 2016 Trimbos Instiute. https://www.trimbos.nl/actueel/nieuws/bericht/jaarbericht-2016-van-de-nationale-drug-monitor-verschenen. Accessed 4 July 2019

[4] Nienhuis KA, Van der Lely NA, Van Hoof JJ (2017). Ten years of alcohol intoxication in adolescents and treatment in pediatric departments in Dutch hospitals. J Addict Res.

[5] Armstrong TD, Costello EJ (2002). Community studies on adolescent substance use, abuse, or dependence and psychiatric comorbidity. J Consult Clin Psychol.

[6] Couwenbergh C, van den Brink W, Zwart K, Vreugdenhil C, van Wijngaarden-Cremers P, van der Gaag RJ (2006). Comorbid psychopathology in adolescents and young adults treated for substance use disorders: a review. Eur Child Adolesc Psychiatry.

[7] Skogen JC, Sivertsen B, Lundervold AJ, Stormark KM, Jakobsen R, Hysing M (2014). Alcohol and drug use among adolescents: and the co-occurrence of mental health problems Ung@Hordaland, a population-based study. Br Med J Open.

[8] Woolfenden S, Dossetor D, Williams K (2002). Children and adolescents with acute alcohol intoxication/self-poisoning presenting to the emergency department. Arch Pediatr Adolesc Med.

[9] Thijssen MA, De Boo GM, Plötz (2018). Single episode of alcohol intoxication in adolescents has no long-term psychosocial effects. Cogent Med.

[10] De Visser MDC, Van Zanten E, Van der Lely NA (2013). Alcohol intoxication and risk factors in adolescents. Addict Sci Clin Pract.

[11] Achenbach TM, Edelbroch C (1979). The child behaviour profile: II. Boys aged 12-16 and girls aged 6-11 and 12-16. J Consult Clin Psychol.

[12] Ormel J, Raven D, van Oort F, Hartman CA, Reijneveld SA, Veenstra R, Vollebergh WA, Buitelaar J, Verhulst FC, Oldehinkel AJ (2015). Mental health in Dutch adolescents: a TRAILS report on prevalence, severity, age of onset, continuity and co-morbidity of DSM disorders. Psychol Med.

[13] Verelst S, Moonen PJ, Desruelles D, Gillet JB (2012). Emergency department visits due to alcohol intoxication: characteristics of patients and impact on the emergency room. Alcohol Alcohol.

[14] Adam A, Faouzi M, Yersin B, Bodenmann P, Deappen JB, Bertholet N (2016). Women and men admitted for alcohol intoxication at an emergency department: alcohol use disorders, substance use and health and social status 7 years later. Alcohol Alcohol.

[15] Neves P, Neuffer N, Yersin B (2011). Massive alcoholic poisoning in the emergency department: how many, who, what and how?. Rev Méd Suisse.

[16] Matali Costa JL, Serrano Troncoso E, Pardo Gallego M (2012). Profile of adolescents seen in emergency departments with acute alcohol poisoning. An Pediatr (Barc).

[17] Brady KT, Randall CL (1999). Gender differences in substance use disorders. Psychiatr Clin N Am.

[18] Squeglia LM, Sorg SF, Schweinsburg AD, Wetherill RR, Pulido C, Tapert SF (2012). Binge drinking differentially affects adolescent male and female brain morphometry. Psychopharmacology.

[19] Liang W, Chikritzhs T (2013). Age at first use of alcohol and risk of heavy alcohol use: a population-based study. Biomed Res Int.

[20] Morean ME, Kong G, Camenga DR, Cavallo DA, Connell C, Krishnan-Sarin S (2014). First drink to first drunk: age of onset and delay to intoxication are associated with adolescent alcohol use and binge drinking. Alcohol Clin Exp Res.

[21] Micheau PA, Belanger R, Mazur A, Hadjipanayis A, Ambresin AE (2020). How can primary care practitioners address substance use by adolescents? A position paper of the European academy of Paediatrics. Eur J Pediatr.

[22] Polanczyk G, de Lima MS, Horta BL, Biederman J, Rohde LA (2007). The worldwide prevalence of ADHD: a systematic review and metaregression analysis. Am J Psychiatr.

[23] Willcutt EG (2012). The prevalence of DSM-IV attention-deficit/hyperactivity disorder: a meta-analytic review. Neurotherapeutics.

[24] Lee SS, Humphreys KL, Flory K, Liu R, Glass K (2011). Prospective association of childhood attention-deficit/hyperactivity disorder (ADHD) and substance use and abuse/dependence: a meta-analytic review. Clin Psychol Rev.

[25] Pedersen SL, Walther CA, Harty SC, Gnagy EM, Pelham WE, Molina BS (2016). The indirect effects of childhood attention deficit hyperactivity disorder on alcohol problems in adulthood through unique facets of impulsivity. Addiction.

[26] Ozgen H, Spijkerman R, Noack M, Holtmann M (2020). International consensus statement for the screening, diagnosis, and treatment of adolescents with concurrent attention-deficit/hyperactivity disorder and substance use disorder. Eur Addict Res.

[27] Baxter AJ, Brugha TS, Erskine HE, Scheurer RW, Vos T, Scott JG (2015). The epidemiology and global burden of autism spectrum disorders. Psychol Med.

[28] De Alwis D, Agrawal A, Reiersen AM, Constantino JN, Henders A, Martin NG, Lynskey MT (2014). ADHD symptoms, autistic traits, and substance use and misuse in adult Australian twins. J Stud Alcohol Drugs.

[29] Edwards AC, Joinson C, Dick DM, Kendler KS, Macleod J, Munafò M, Hickman M, Lewis G, Heron J (2014). The association between depressive symptoms from early to late adolescence and later use and harmful use of alcohol. Eur Child Adolesc Psychiatry.

[30] Central Bureau of Statistics (2018) Gezondheid en zorggebruik; persoonskenmerken Depressie. Available from: https://opendata.cbs.nl/statline/. Accessed at 4 July 2019

[31] Terra MB, Figuera I, Barros HM (2004). Impact of alcohol intoxication and withdrawal syndrome on social phobia and panic disorder in alcoholic inpatients. Revista do Hospital das CLinicas da Faculdade de Medicina da Universidade de Sao Paulo.

[32] Fjord S, Ranta K, Kaltiala-Heino R, Marttunen M (2011). Associations between social phobia and general anxiety with alcohol and drug use in a community sample of adolescents. Alcohol Alcohol.

[33] Wu P, Bird HR, Liu X, Duarte CS, Fuller C, Fan B, Shen S, Canino GJ (2010). Trauma, posttraumatic stress symptoms, and alcohol-use initiation in children. J Stud Alcohol Drugs.

[34] Read JP, Radomski S, Borsari B (2015). Associations among trauma, posttraumatic stress, and hazardous drinking in college students: considerations for intervention. Curr Addict Rep.

[35] Read JP, Colder CR, Merrill JE, Ouimette P, White J, Swartout A (2012). Trauma and posttraumatic stress symptoms influence alcohol and other drug problem trajectories in the first year of college. J Consult Clin Psychol.

[36] Stappenbeck CA, Bedard-Gilligan M, Lee CM, Kaysen D (2013). Drinking motives for self and others predict alcohol use and consequences among college women: the moderating effects of PTSD. Addict Behav.

[37] Hopfer C, Salomonsen-Sautel S, Mikulich-Gilbertson S, Min SJ, McQueen M, Crowley T, Young S, Corley R, Sakai J, Thurstone C, Hoffenberg A, Hartman C, Hewitt J (2013). Conduct disorder and initiation of substance use: a prospective longitudinal study. Am Acad Child Adolesc Psychiatry.

[38] De Wit DJ, Adlaf EM, Offord DR, Ogborne AC (2000). Age at first alcohol use: a risk factor for the development of alcohol disorders. Am J Psychiatr.

[39] Grant BF, Dawson DA (1997). Age at onset of alcohol use and its association with DSM-IV alcohol abuse and dependence: results from the National Longitudinal Alcohol Epidemiologic Survey. J Subst Abus.

[40] Diagnostic and Statistical Manual of Mental Disorders 5 (DSM-5) 47(American Psychiatric Association. 2013)

[41] Martinotti G, De Risio L, Vannini C, Schifano F, Pettorruso M, Di Giannantonio M (2020). Substance related exogenous psychosis: a post-modern syndrome. CNS Spectrums.

